# A Comprehensive Review: Unraveling the Role of Inflammation in the Etiology of Heart Failure

**DOI:** 10.1007/s10741-025-10519-w

**Published:** 2025-05-14

**Authors:** Diana Roman-Pepine, Adela Mihaela Serban, Roxana-Denisa Capras, Cristina Mihaela Cismaru, Adriana Gabriela Filip

**Affiliations:** 1 Department of Anatomy and Embryology, Iuliu Hatieganu University of Medicine and Pharmacy, 400348 Cluj-Napoca-Napoca, Romania; 2 Cardiology Department, Heart Institute Niculae Stăncioiu, 19–21 Motilor Street, 400001 Cluj-Napoca- Napoca, Romania; 3 5 Th Department of Internal Medicine, Iuliu Hatieganu University of Medicine and Pharmacy, 400348 Cluj-Napoca-Napoca, Romania; 4 Department of Infectious Diseases, Iuliu Hatieganu University of Medicine and Pharmacy, 400348 Cluj-Napoca-Napoca, Romania

**Keywords:** Heart Failure, Inflammation, Cytokines, Interleukins, Biomarkers

## Abstract

Heart failure (HF) remains a leading cause of morbidity and mortality worldwide, with inflammation playing a pivotal role in its pathogenesis. This comprehensive review aims to elucidate the intricate mechanisms by which inflammation contributes to the development and progression of HF. The review synthesizes current research on the involvement of both innate and adaptive immune responses in HF, highlighting the roles of cytokines, chemokines, and other inflammatory mediators. Recent studies have demonstrated that chronic inflammation, driven by factors such as oxidative stress, neurohormonal activation, and metabolic disturbances, leads to adverse cardiac remodeling and impaired myocardial function. The review explores how systemic inflammation, characterized by elevated levels of inflammatory biomarkers like C-reactive protein (CRP) and interleukin-6 (IL-6), correlates with HF severity and outcomes. Additionally, it discusses the impact of comorbid conditions such as diabetes, obesity, and hypertension on inflammatory pathways and HF risk. The review also delves into the therapeutic implications of targeting inflammation in HF. Despite mixed results from early clinical trials, emerging evidence suggests that anti-inflammatory therapies offer benefits in specific HF phenotypes. The potential of novel therapeutic strategies, including the use of biologics and small molecule inhibitors, is examined in the context of their ability to modulate inflammatory responses and improve clinical outcomes.

## Introduction

Heart failure (HF) is a complex clinical syndrome that develops due to structural or functional impairments of the ventricular ejection or filling [1]. It is a global disease that affects more than 26 million people worldwide and is increasing in prevalence, due to the rising age of the population. Even with substantial progress in treatment and prevention methods, the rates of mortality and morbidity remain elevated, and the quality of life is often compromised [2].

Multiple pathogenic mechanisms are involved in the development of heart failure. These include hemodynamic overload, ventricular dysfunction secondary to ischemia and structural changes in the ventricular architecture. The etiology of HF is diverse, including coronary artery disease, hypertension, valvulopathies and cardiomyopathies.

However, regardless of the underlying cause, inflammation is a common factor in the etiology of heart failure. Inflammatory mediators such as cytokines, chemokines, and adhesion molecules are upregulated in HF, contributing to the progression of the disease [3]. The inflammatory response in HF is an essential instrument but can also cause more harm than good. While it is necessary for tissue repair and healing, excessive inflammation can lead to adverse cardiac remodeling, further myocardial injury, and worsening of HF [4]. Biomarkers, which are released due to myocardial extensive stretching, an imbalance in the formation and degradation of the extracellular matrix, release of cytokines due to a proinflammatory state and, in the final stages, kidney failure, play a key role in identifying the pathogenic mechanism [5].

This review aims to provide a comprehensive overview of the role of inflammation in the etiology of HF. We will discuss the molecular mechanisms of inflammation in HF, examine the inflammatory markers associated with HF, primers of inflammation and consider potential anti-inflammatory therapeutic strategies for HF.

## Mechanisms of inflammation in heart failure

### General Considerations

Inflammation influences heart failure in various ways, from contributing to the development of HF related comorbidities like diabetes and obesity [6], to affecting pathological conditions underlying heart disease such as endothelial dysfunction and atherosclerosis [7]. In addition, heart failure induces sterile inflammation within the heart, triggered by wall stress and signals released by malfunctioning or dead cells [8]. When wall stress increases all cells are subjected to greater biomechanical strain. Mechano-sensitive adhesion proteins, such as integrins and cadherins, transmit mechanical signals between cells and their environment, which can trigger cellular responses like growth, differentiation, and inflammation [9]. When cardiac fibroblasts are mechanically stretched they produce not only more extracellular matrix but also chemokines and activate typical inflammatory pathways. This, consequently activates the recruitment of monocytes by enabling their migration across the endothelium into cardiac tissue [10]. Additionally, mechanical stretching of cardiac fibroblasts (as opposed to cardiomyocytes) leads to the release of IL-1ß, which triggers leukopoiesis in the bone marrow and at extramedullary sites. The cardiac cytokines and other inflammatory mediators that are released not only impact the heart but also affect other organs [11, 12].

In recent times, oxidative stress and inflammation have been recognized as crucial elements in the pathophysiology of heart failure syndrome and potential factors contributing to the progression of heart failure. Reactive oxygen and nitrogen species (ROS/RNS) play a signaling role in a healthy heart, but an excessive and unregulated production of these molecules can lead to oxidative stress and damage to cardiomyocytes. There are several mechanisms that contribute to mitochondrial dysfunction and increased ROS/RNS production, such as activation of neurohormones, pressure and volume overload, and changes in cardiac energy metabolism. Additionally, comorbidities that promote systemic inflammation, such as diabetes, obesity, or sleep apnea, also contribute [13].

In response, ROS/RNS can have harmful effects on cellular components, including mitochondria, which further promotes the generation of free radicals and increases intracellular oxidative stress. This self-perpetuating cycle leads to functional and structural changes in the ventricular matrix that accelerate the progression of heart failure [14].

Multiple studies have demonstrated the potential involvement of several groups of cytokines and chemokines in acute and chronic HF. However, targeting these pathways in early therapeutic trials have produced mixed results. These studies served to highlight the complexity and nuances of how pro-inflammatory pathways contribute to the pathogenesis of HF [15].

#### Specific considerations regarding systolic left ventricular function (HFpEF vs HFrEF)

The limitations of left ventricular ejection fraction (LVEF) as an indicator of systolic LV function are especially pronounced in HF patients, even before clinical symptoms appear. Beyond assessing symptoms and signs, HF diagnosis heavily depends on LVEF as a measure of ventricular cavity function, typically evaluated through 2D echocardiography in clinical settings. Findings from most HF clinical trials suggest that treatment benefits are predominantly observed in patients with reduced LVEF [16, 17]. Present guidelines classify HF patients into distinct phenotypes based on LVEF as follows. Heart Failure with Reduced Ejection Fraction (HFrEF) is often defined by an LVEF below 40%. This phenotype, also known as systolic heart failure, is marked by impaired contractility or pumping function of the heart, leading to the ventricle's inability to eject sufficient blood. Heart Failure with Mid-Range Ejection Fraction (HFmrEF) who falls in the intermediate range, often with an LVEF between 40 and 49%. HFmrEF represents a transitional group that shares features of both HFrEF and HFpEF [18]. Patients in this category exhibit overlapping clinical and pathophysiologic traits, and because more research is needed to clarify their optimal management strategies and pathophysiology it is beyond the scope of our review. Heart Failure with Preserved Ejection Fraction (HFpEF) is typically characterized by an LVEF of 50% or higher. Despite a normal or near-normal ejection fraction, these patients have diastolic dysfunction where the heart's relaxation and filling phases are impaired. HFpEF is often seen in older adults and is associated with comorbid conditions such as hypertension, obesity, diabetes, and atrial fibrillation [18].

Starting from the complexity of diagnosing heart failure with preserved ejection fraction (HFpEF), two algorithms were proposed (H2 FPEF Score and ESC HFA-PEFF Algorithm). A study by Selvaraj et al. applied both algorithms to a large community-based cohort (the ARIC study) and found that although both scores similarly predicted adverse outcomes such as HF hospitalizations and death, there were significant differences in risk classification between them. This observation underscores the necessity for direct validation of these tools against invasive diagnostic methods and highlights the potential for advanced techniques, such as machine learning, to further refine HFpEF diagnosis. In conclusion, while current diagnostic algorithms offer valuable frameworks for assessing HFpEF, further research is needed to standardize their use, improve diagnostic accuracy, and ultimately tailor management strategies for this heterogeneous patient population [19].

Besides the difference in diagnosing HFrEF and HFpEF, some studies also shown a difference in the role inflammation plays in the development of HF depending on the phenotype.

For heart failure with preserved ejection fraction (HFpEF), a new paradigm suggests that a systemic pro-inflammatory state caused by comorbidities leads to microvascular endothelial cell inflammation, which then triggers HFpEF-specific concentric cardiac remodeling and dysfunction [3]. In contrast, for heart failure with reduced ejection fraction (HFrEF), inflammation is driven by direct cardiomyocyte damage from events like myocardial ischemia, leading to eccentric cardiac remodeling and dysfunction [8].

Irrespective of the underlying phenotype, a common finding across studies is that inflammation is more highly prevalent in HFpEF and is linked to worsened symptoms and prognosis [20].

A prospective, single‐center study examined 56 symptomatic HFpEF patients stratified by left ventricular ejection fraction (LVEF) into two cohorts: one with LVEF between 50 and 60% and another with LVEF greater than 60%. Using a multimodal approach (including echocardiography, cardiac magnetic resonance imaging and myocardial biopsy) the authors comprehensively assessed cardiac structure and function. The study particularly focused on differences in ventricular volumes, myocardial fibrosis, contractility, and hemodynamic responses at rest and during handgrip exercise to unravel distinct pathophysiologic subphenotypes within HFpEF. Patients with LVEF between 50 and 60% demonstrated larger ventricular dimensions, greater amounts of myocardial fibrosis, reduced contractility, and impaired ventriculo‐arterial coupling. In contrast, those with LVEF > 60% exhibited smaller ventricles preserving stroke volume through higher ejection fractions, yet they showed a hypercontractile state marked by increased systolic and diastolic stiffness [21].

Popovic et al. assessed HFpEF patients using a slightly different stratification, comparing those with LVEF < 65% (covering 50%–65%) against those with LVEF ≥ 65%, and included both an invasive cohort with HFpEF and a community-based non-invasive cohort with healthy controls. Their findings similarly demonstrated that patients with higher ejection fractions have smaller left ventricles, as evidenced by a leftward-shifted end-diastolic pressure–volume relationship and increased diastolic stiffness, indicative of chamber contracture. Despite the different LVEF cutoff points used in each study, both articles converge on the observation that a higher ejection fraction in HFpEF correlates with adverse structural remodeling and a stiffened ventricle, which partially explain the diminished response to conventional neurohormonal therapies [22].

Comparative studies of HFpEF and HFrEF cohorts have consistently shown that inflammation tends to be more prevalent in HFpEF compared to HFrEF, possibly indicating a distinct pathophysiology [23]. The BIOSTAT-CHF study conducted a biomarker analysis that revealed specific biological pathways related to inflammation that were unique to HFpEF, as opposed to HFrEF. These biomarker profiles of HFrEF are associated with cellular proliferation and metabolism, while those specific to HFpEF are linked to inflammation and the reorganization of the extracellular matrix [24].

This result was corroborated in a second cohort and mirrors an earlier analysis from the TIME-CHF study, which found that biomarkers related to inflammation, such as IL-6 and hsCRP, were upregulated in HFpEF compared to HFrEF but with no important difference regarding prognostic [25].

Comorbidity-driven systemic microvascular inflammation has emerged to characterize the link between metabolic stressors, including diabetes, obesity, insulin resistance, non-alcoholic fatty liver disease, and persistent inflammation [3]. Over half of the participants in the 2018 HFpEF trial (RELAX) exhibited elevated levels of C-reactive protein [26]. This prolonged inflammatory condition can set the stage for cardiac remodeling which ultimately result in heart failure with preserved ejection fraction [27].

In addition to the inflammation state observed in HFpEF compared to HFrEF, inflammation is also linked to more severe cardiac structural and functional abnormalities in HFpEF patients. A proteomic analysis involving 228 HFpEF patients from the multicenter PROMIS-HFpEF study (Prevalence of Microvascular Dysfunction in Heart Failure with Preserved Ejection Fraction) examined 47 proteins involved in inflammatory pathways and discovered that systemic inflammation was correlated with a higher number of comorbidities, diastolic dysfunction, and right ventricular dysfunction. These were highlighted using echocardiography parameters such as mitral E velocity, E/e′ ratio, and tricuspid regurgitation velocity, tricuspid annular plane systolic excursion and right ventricular free wall strain [28].

While measures of left ventricular and left atrial volume overload, as well as indicators of LV diastolic function, independently predict mortality, left ventricular ejection fraction (LVEF) does not significantly enhance mortality risk stratification. Several large-scale studies involving patients with various ejection fraction values have demonstrated similar mortality rates across the entire LVEF spectrum [29]. Additionally, epidemiologic data reveal a U-shaped relationship between mortality and LVEF in those with LVEF ≥ 65%, where mortality rates are comparable to those observed in patients with heart failure with reduced ejection fraction [30]. These findings indicate that classifying systolic heart failure based solely on ejection fraction not consistently yield an accurate risk assessment. Notably, global longitudinal strain (GLS) has proven to be a stronger predictor of cardiovascular outcomes than LVEF, despite its sensitivity to factors such as age, sex, and loading conditions [31].

Therefore, novel methods to characterize the distinct phenotypes of heart failure are essential for deepening our understanding of its pathophysiology and for identifying promising new therapeutic targets. In this context, we will address the diverse mechanisms that trigger inflammation and their impact on heart failure progression. Furthermore, systemic inflammation is a predictor of all-cause mortality. The activation of immune pathways is triggered by neurohormonal systems such as the renin–angiotensin–aldosterone system, the sympathetic nervous system, and the natriuretic peptides system. Therefore, the promotion of inflammation by these mechanisms and the subsequent development of heart failure suggest that the immune system plays an essential role in the progression of heart failure [32].

### Inflammatory Markers associated with Heart Failure

#### High Sensitive C‑reactive protein

**Fig. 1:**
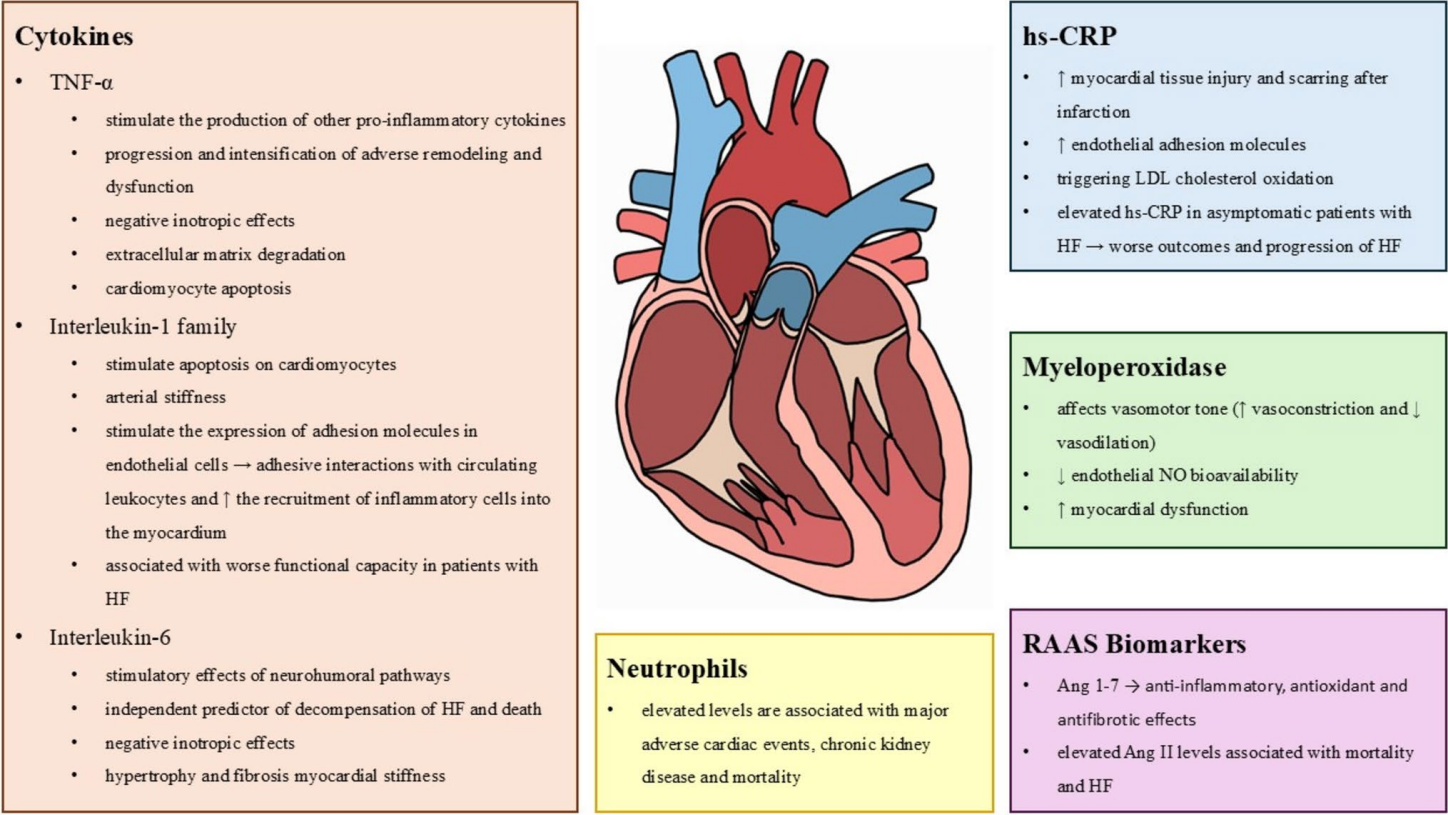
Central Illustration—The most studied markers of inflammation in heart failure

Hight sensitive C-reactive protein (hs-CRP) is a cytokine associated with inflammation that is primarily produced by hepatic cells as a response to interleukin-6 (IL-6) signaling (Fig. 1). It has been identified as a potential indicator for negative outcomes in patients with heart failure. In a study by Vasan and others, the relationship between hs-CRP and other inflammation markers and the occurrence of HF events was examined in patients without HF or ischemic heart disease at the start of the study. After adjusting for traditional risk factors, it was found that individuals with baseline hs-CRP levels equal or higher than 5 mg/dl had an elevated risk of HF. This risk was further increased with the release of other inflammation markers in addition to hs-CRP [33]. A similar analysis was conducted by Pellicori and colleagues in patients with chronic HF and it was found that higher baseline levels of hs-CRP independently predicted a higher rate of all-cause and cardiovascular mortality [34]. It is still uncertain whether targeting the inflammatory pathways that determine hs-CRP levels can improve clinical outcomes in HF patients, especially when considering the side effects of these therapies. It also remains to be seen whether some of the benefits of established HF therapies are mediated by reductions in hs-CRP levels [35].

Numerous studies have explored the impact of various pharmacological and non-pharmacological therapies on plasma hs-CRP levels in patients with coronary heart disease, but there is limited information regarding heart failure patients. While lifestyle changes and exercise have been shown to lower hs-CRP [36], statins and aspirin remain the primary treatments for elevated hs-CRP [37]. Additionally, blocking the renin-angiotensin system with ACE inhibitors has recently demonstrated anti-inflammatory effects. Despite the known role of inflammation in heart failure progression, there is currently no clinical data on the benefits of reducing inflammatory activity in this condition [38].

The findings of a study published in Circulation indicate a direct link between increased plasma hs-CRP levels and the progression of heart failure (HF) [39]. Elevated plasma CRP is correlated with a more unfavorable hemodynamic and neurohormonal profile, as well as a lower quality of life. hs-CRP serves as a predictor of negative clinical outcomes, regardless of whether the etiology is ischemic or nonischemic, and independent of other adverse outcome predictors [39]. Therefore, measuring CRP could offer additional prognostic insights, complementing global risk assessment in HF patients.

#### Inflammatory cytokines

Heart failure is marked by an imbalance between pro-inflammatory and anti-inflammatory cytokines. A higher level of pro-inflammatory cytokines is often associated with a more severe state of HF, including cardiogenic shock [40].

These cytokines, primarily released by neutrophils, contribute to the death of cardiomyocytes and the activation metalloproteinases. This leads to changes in the structure of the myocardium that has negative impacts on its function. Several pro-inflammatory and anti-inflammatory cytokines, including IL-1, IL-6, IL-8, IL-18, IL-1RA, and IL-33, have been found in the bloodstream of patients diagnosed with HF [41] (Table 1).

**Table 1:** Cytokines implicated in heart failure [42]

Cytokines	Functions	Participation in pathogenesis	Prognostic value	Identify high-risk patients	Potential therapeutic target	Diagnostic value
TNF-α	Induce inflammatory genes expression and apoptosis, release proinflammatory cytokines, promote adverse remodeling	Yes	Yes	No	No	Yes
IL-1	Induce negative inotropic effect through impairing β-adrenergic responsiveness and disturbing calcium handling	Yes	Yes	No	No	No
IL-6	Pleiotropic proinflammatory responses	Yes	Yes	No	Yes	No
IL-10	Inhibits proinflammatory cytokines secretion, block ROS release, modulate TNF-a-mediated responses	No	No	No	Yes	No

### Tumor Necrosis Factor Alpha

Tumor necrosis factor alpha (TNF-α) is a widely researched proinflammatory cytokine in HF. It can be produced by various cell types in the heart, including activated macrophages, cardiomyocytes, vascular cells, and mastocytes cells [43]. TNF-α, which can exist in membrane-bound or cytosolic forms, performs its functions by binding to TNFR1 or TNFR2 receptors on the cell membrane [44].

TNF-α has been implicated in several detrimental effects on heart structure and function. These include negative inotropic effects due to calcium homeostasis disruption, upregulation of other inflammatory molecules, including the induction of inducible NO synthase, enhancement of oxidative stress leading to mitochondrial DNA damage, promotion of apoptosis and extracellular matrix degradation, and increased microvascular endothelial permeability and activation, which enhances endothelial-leukocyte interactions [45].

**Fig. 2:**
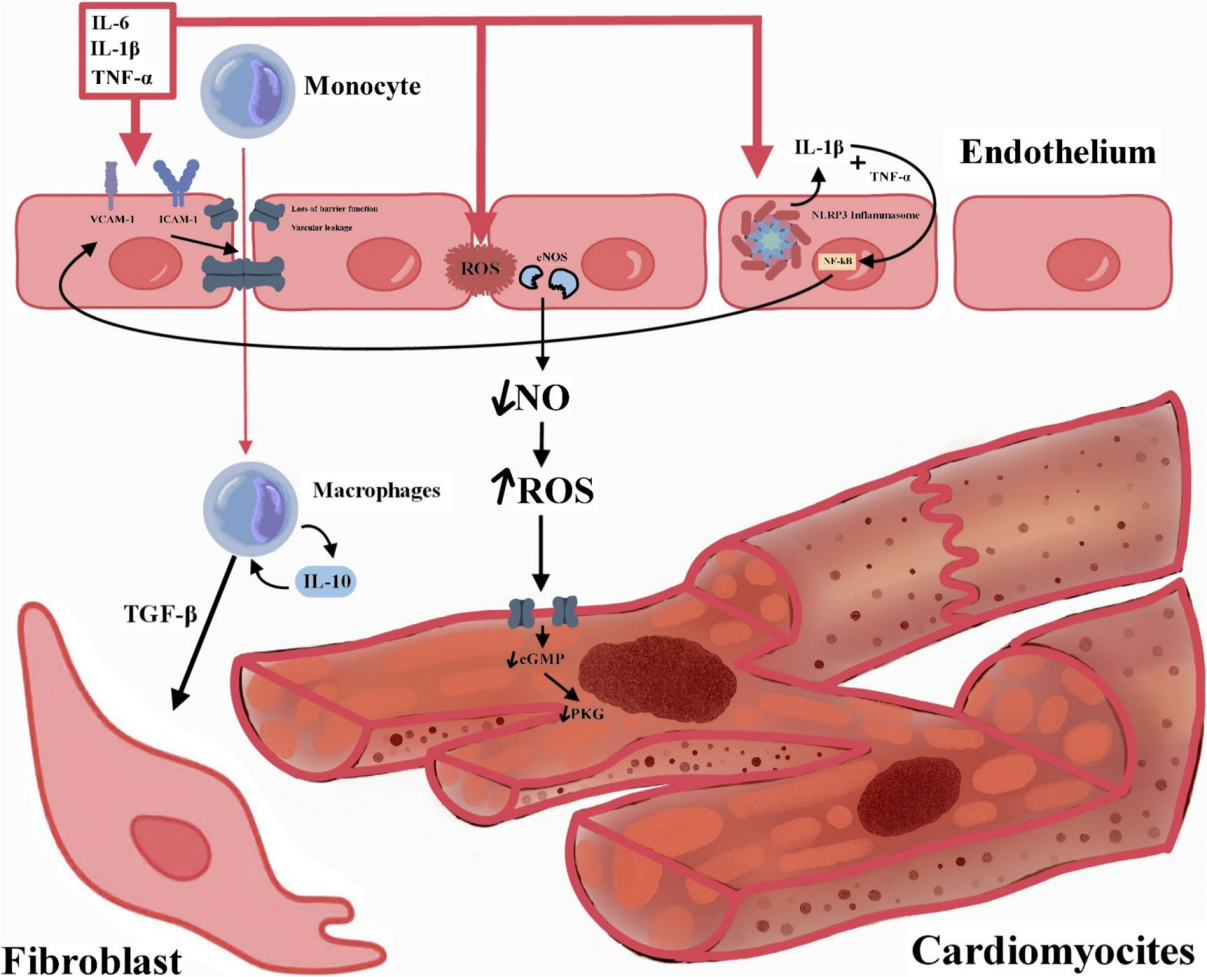
Endothelial Dysfunction in Heart Failure. IL – interleukin; TGF-β—transforming growth factor beta; ROS – reactive oxygen species; TNFα – tumor necrosis factor-α; NF-kB – nuclear factor kappa-light-chain-enhancer of activated B cells; NLRP3 inflammasome – NOD-like receptor protein 3 inflammasome; cGMP – cyclic guanosine monophosphate; PKG – protein kinase G; ICAM-1 – Intercellular adhesion molecule 1; VCAM −1 – Vascular cell adhesion protein 1.

**Fig. 3:**
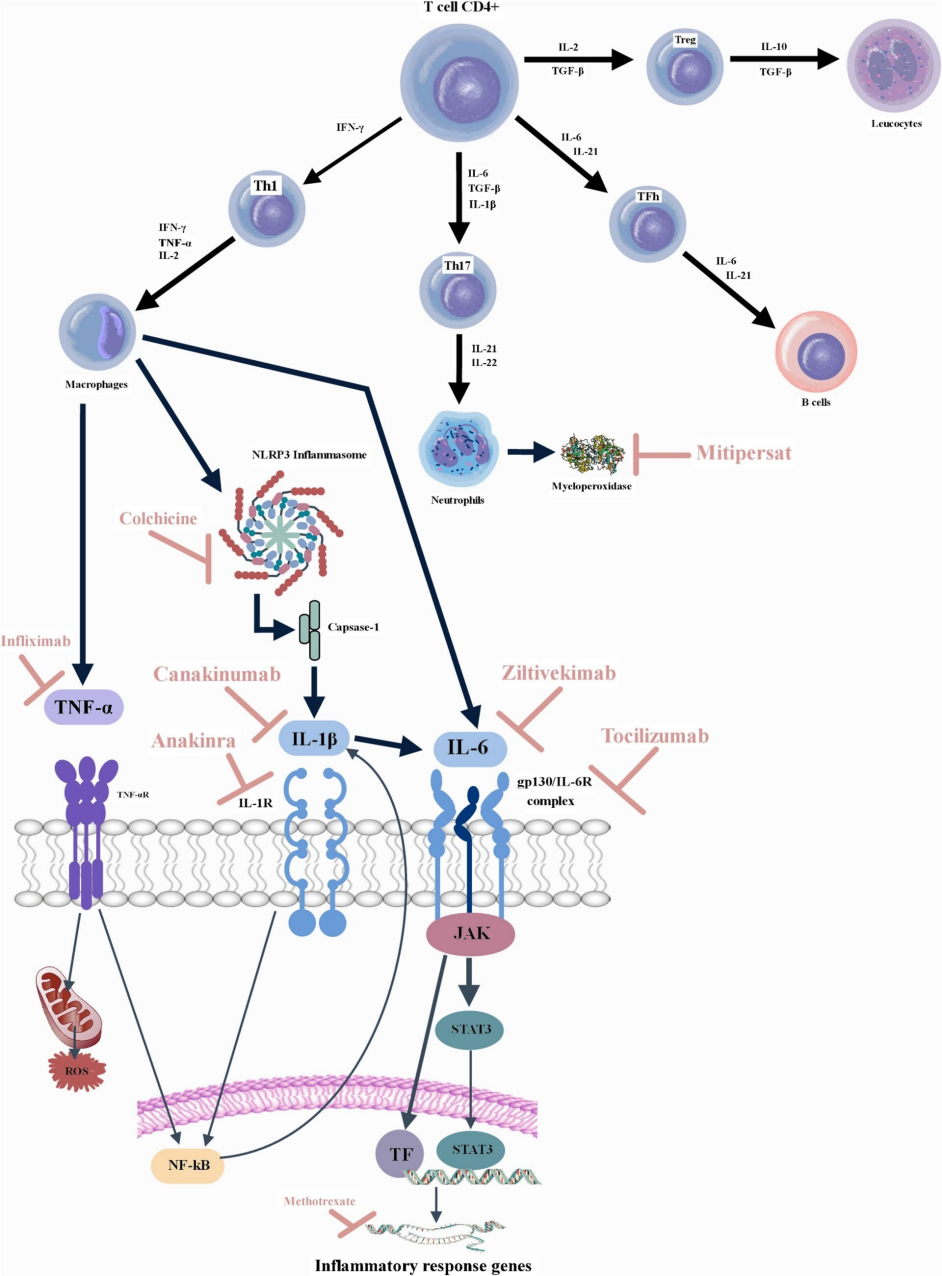
Inflammatory pathways and targeted anti-inflammatory therapeutics. CD – cluster of differentiation; IL – interleukin; IFN-γ – interferon gamma; TGF-β—transforming growth factor beta; IL-1R – interleukin-1 receptor; IL-6R – interleukin-6 receptor; ROS – reactive oxygen species; TF – transcription factor; TNFα – tumor necrosis factor-α; TNFαR – tumor necrosis factor-α receptor; JAK – Janus kinase; NF-kB – nuclear factor kappa-light-chain-enhancer of activated B cells; NLRP3 inflammasome – NOD-like receptor protein 3 inflammasome; STAT3—signal transducer and activator of transcription 3

In macrophages, TNF-α could stimulate the production of other pro-inflammatory cytokines with pro-apoptotic, negative inotropic, and matrix-degrading properties, and increase the expression of inducible nitric oxide synthase (iNOS) [45, 46]. In fibroblasts, TNF-α could disrupt the equilibrium between matrix metalloproteinases (MMPs) and their inhibitors, leading to degradation of the extracellular matrix (ECM) [47]. At the level of the vascular system, TNF-α can enhance permeability by modulating endothelial cyclooxygenase-2, and induce the expression of endothelial adhesion molecules, such as intercellular adhesion molecule (ICAM)−1 and vascular cell adhesion molecule (VCAM)−1, thereby enhancing adhesive interactions between circulating leukocytes and the endothelial lining (Fig. 2) [48, 49]. This could result in neutrophils or pro-inflammatory monocytes being trapped in cardiac microcirculation, contributing to tissue damage and cardiac dysfunction (Fig. 1, Tabel 1, Fig. 3).

In addition to its role in the progression and intensification of adverse remodeling and dysfunction, TNF-α is also suggested to have protective effects on injured or stressed heart muscle cells. In a non-reperfused myocardial infarction model, the complete removal of TNF receptors (TNFRs) was linked to an increase in infarct size, implying that TNF-α signaling could transmit cytoprotective signals [50]. In a genetic model of cardiomyopathy caused by the loss of desmin, TNF-α was found to have cytoprotective effects by encouraging the formation of an alternate cytoskeletal network that prevents the decline of cardiac function [51]. In isolated rat hearts, a low dose of TNF-α improved hemodynamics, with the beneficial effects attributed to TNF-α’s inhibitory impact on cardiomyocyte calcium influx, potentially reducing intracellular calcium overload [52].

The contradictory findings suggesting both protective and harmful effects of TNF-α in vivo reflect dose-dependent effects or differences in the balance between TNFR1 and TNFR2 signaling in various cell types and experimental models. It has been proposed that the harmful effects of TNF-α on cardiac remodeling be mediated through TNFR1 signaling, while TNFR2 actions be beneficial. However, the cell-specific actions of TNFR signaling in vivo and their role in mediating cardiac remodeling have not been thoroughly explored [43] (Table 1).

### Interleukin-1 family

The IL-1 family, which consists of 11 cytokine members and 10 receptors, includes IL-1α/IL-1β, IL-18, and the IL-33/ST2 axis. These are the most extensively studied within the cardiovascular system. A significant amount of experimental data suggests that the IL-1 family members could have a crucial role in the progression of heart failure and the development of systolic dysfunction [53]. Many experimental studies show a significant role of IL-1 signaling in the progression of heart dysfunction and ventricular remodeling that leads to heart failure [54].

Similar to TNF-α, IL-1 suppress the function of systolic cardiomyocytes, potentially through disruption of calcium handling or suppression of β-adrenergic responses [55]. IL-1 can also stimulate apoptosis on cardiomyocytes and can induce the mobilization and activation of leukocytes, thereby stimulating subsequent inflammatory responses [56]. IL-1 might stimulate the expression of adhesion molecules in endothelial cells, promoting adhesive interactions with circulating leukocytes and increasing the recruitment of inflammatory cells into the myocardium [57]. The matrix degradation driven by IL-1 ultimately activate fibroblast-mediated matrix protein synthesis, leading to increased fibrosis through the upregulation of fibrogenic growth factors [58]. It might increase arterial stiffness and microvascular inflammation, contributing to the pathogenesis of heart failure with preserved ejection fraction (HFpEF) [59] (Fig. 1, Fig. 3).

Pharmacological interventions targeting IL-1 pathways have demonstrated protective effects in experimental models. The administration of recombinant IL-1Ra (anakinra) protected the myocardium from harmful remodeling in rodent models following HF secondary to myocardial infarction but also in cases of non-ischemic HF [60]. Treatment with anti-IL-1β antibodies has proved effective in heart failure models. In both diabetic and non-diabetic rats with heart failure following a myocardial infarction, the administration of the anti-IL-1β antibody gevokizumab reduced the ventricular dimensions and improved its function (measured by echocardiography), decreasing hypertrophy and fibrosis [61].

### Interleukin-6

IL-6 is a member of the gp130 family of cytokines. This family also encompasses several other cytokines, including IL-11, leukemia inhibitory factor (LIF), cardiotrophin-1, and oncostatin-M, which have been associated with the development of cardiovascular diseases. These cytokines transmit signals via the shared signaling receptor subunit gp130, leading to the activation of Janus kinases (JAKs) and subsequent phosphorylation of STAT3 [62] (Fig. 1, Fig. 3).

In experimental models of cardiac injury and heart failure, IL-6 is consistently elevated. It is expressed by cardiomyocytes, infiltrating mononuclear cells, and fibroblasts [63]. In heart failure where there is pressure overload, the induction of IL-6 is accompanied by an upregulation of the IL-6 receptor (IL-6Rα) [64]. The increased expression of IL-6 reflect the stimulatory effects of neurohumoral pathways, Toll-like receptor (TLR) agonists, or other pro-inflammatory cytokines (such as TNF-α and IL-1) on myocardial cells or infiltrating leukocytes. During the progression of heart failure, negative regulatory mechanisms be activated to limit the expression of IL-6 and other pro-inflammatory cytokines. One such mechanism could be the degradation of IL-6 mRNA by the RNAse regnase-1, which has been suggested to restrain IL-6 expression and its pro-inflammatory actions in the pressure-overloaded myocardium [65].

Experimental studies on the role of IL-6 in heart failure have conflicting results, depending on the model, the pathophysiological context, and the type of interventions used to study the effects of IL-6 signaling. Most evidence suggests that IL-6 signaling primarily exacerbates inflammation and ventricular dysfunction. Persistent gp130/STAT3 signaling has been shown to enhance inflammation in remodeling post myocardial infarction [66]. Furthermore, in high pressure situations, the genetic loss of IL6 has been found to improve cardiac function and reduce hypertrophy, likely due to the abrogation of CaMKII-dependent effects on cardiomyocytes [67]. However, other studies using similar genetic approaches found no significant effects of IL6 deletion on pressure-overload [68].

There is substantial evidence indicating that IL-6 has an important role in the post-inflammatory hepatic acute phase response. Given this evidence, IL-6 has been viewed as a promising therapeutic target for many inflammation-related conditions, including HFpEF [69]. Tocilizumab, an antibody that neutralizes the IL-6 receptor, has been approved as an effective treatment for patients with moderate to severe rheumatoid arthritis and temporal arteritis, as well as for treating the cytokine release syndrome associated with CAR-T cell therapies. Experimental evidence showing the role of IL-6 signaling in the development of heart failure suggests that tocilizumab treatment could also be beneficial for patients with heart failure [70].

## Immune cells

### Neutrophils

Neutrophils have a significant role in the progression of cardiovascular diseases, contributing to tissue damage and cardiac remodeling. In patients with severe heart failure, the inflammation response derived from neutrophils has been studied in various states, at rest, post-stimulation, and following suppression by immunosuppressive drugs [71] (Fig. 1, Fig. 3).

Numerous studies have explored the significance of the neutrophil-to-leukocyte ratio (NLR), which has been found to have a strong correlation with heart failure, increased mortality, major cardiovascular events, HF hospitalizations, and chronic kidney disease in elderly patients [72].

### Macrophages

Various types of macrophages with distinct roles and origins have been identified in cardiomyocytes, some of which are protective while others can be harmful [73]. Resident macrophages shift to a more healing-oriented state by reducing the expression of IL-6, TNF-α, and MMP9, influenced by external signals like IL-10 from regulatory T cells [74]. This shift results in cardiac macrophages that produce TGF-β and VEGF, promoting fibrosis and new blood vessel formation, along with other factors like myeloid-derived growth factor. When injury occurs, cytokines and chemokines attract monocytes that turn into Ly6 C high macrophages, which are crucial for removing dead cells and managing inflammatory signals. The transition from inflammation to repair is marked by a reduction in neutrophils and the emergence of Ly6 C low macrophages with lower inflammatory cytokine production [75] (Fig. 3).

Macrophage presence has been noted in myocardial biopsies from patients with HFpEF, playing a role in the disease’s pathophysiology [76]. Classical monocytes (Mon1) primarily produce cytokines IL-1β, IL-6, and MCP-1, intermediate monocytes (Mon2) generate the anti-inflammatory cytokine IL-10, while nonclassical monocytes (Mon3) trigger cytokine production in response to viral infections rather than bacterial ones. Mon2 levels rise in heart failure and are associated with the NYHA classification [77].

### Natural Killer Cells

Natural killer (NK) cells are the largest group within the innate lymphoid cell family, that does not have the antigen receptors present in classical T and B cells of the adaptive immune system. They are crucial for tissue repair and maintaining homeostasis and they influence immune cell behavior directly through receptor-ligand interactions or indirectly by secreting cytokines, lysing auto aggressive T cells, and promoting the maturation of monocytes and dendritic cells [78]. They are vital in containing viral infections and reducing eosinophilic infiltration in myocarditis mouse models. Additionally, NK cells help prevent cardiac fibrosis by limiting collagen production in cardiac fibroblasts and preventing the buildup of certain inflammatory cell populations in the heart [79].

A persistent reduction in NK cells was linked to mild cardiac inflammation, while patients with normalized NK cell levels showed minimal to no cardiac inflammation. Impaired NK cell cytolytic function was associated with baseline IL-6 levels in PBMCs of heart failure patients. Additionally, a decrease in NK cells was noted in coronary artery and ischemic heart disease [80].

### Mast Cells

Mast cells (MC) are noncirculating immune cells, pluripotent leukocytes to be more precise, that mature in target tissues secondary to the presence of the c-kit ligand stem cell factor from bone marrow-derived precursors. Originally identified as primary effector cells in various allergic disorders, these immune cells are now believed to also play roles in certain autoimmune disorders, responses to infections and growing evidence indicates that mast cells also contribute to tissue remodeling and the progression of cardiovascular diseases [81].

They produce and secrete a variety of proinflammatory and vasoactive mediators like TNF-α, proteases (tryptase, chymase, and stromelysin), histamine and transforming growth factor β (TGF-β) which play a role in the activation of matrix metalloproteinases, contributing to fibrosis, stiffening and remodeling in cardiovascular disorders, and, if this process continues end in heart failure [82]. Not only do they initiate the pro-fibrotic processes, but hey can also be activated by the mechanical stress caused by tissue fibrosis [83]. Although this has not been directly proven for human cardiac MC, evidence suggests that in rats, cardiac MC are more sensitive to mechanical stress compared to peritoneal or pleural MC [84].

Patients with congestive heart failure with left ventricular assist devices (LVAD) showed increased stem cell factor and c-kit gene expression and a higher number of mast cells after ventricular unloading [85]. Akgul et al. have shown that cardiac MC are more numerous in patients with cardiomyopathy compared to normal myocardium. Additionally, a further increase in mast cells occurs following long-term mechanical support [86]. Consequently, they discovered that after long-term LVAD support (more than 40 days), there was a further increase in MC numbers compared to patients who received short-term support. This subsequent increase is primarily in chymase-negative mast cells and is associated with reduced fibrosis. This suggests a transition from a pro-fibrotic to an anti-fibrotic MC phenotype [86].

Another implication of MC has been proven to be in post-heart transplant patients. Mast cells quickly increased and degranulated over several months in some post-transplant hearts. The density of mast cells and their secretory products correlated with the extent of fibrosis. Patients with more mast cells experienced more severe rejection reactions. The appearance of mast cells as early as the first week suggested that activated mast cells, along with interactions with other inflammatory cells, play an important role in the rejection process. Mast cell degranulation appears to be a key factor in heart transplant fibrosis and rejection. These findings have therapeutic implications if the level of mast cell infiltration can be monitored and reduced in the first two weeks after cardiac transplantation [87].

### T Cells

During cardiac inflammation, endothelial cells (ECs) upregulate adhesion molecules that facilitate T-cell interactions and extravasation. The initial stage of T-cell transportation is primarily mediated by endothelial selectins, responsible for T-cell rolling on the endothelium, an initial contact that also helps T cells encounter chemokines on the EC surface, necessary for T-cell integrin activation. Activated T-cell integrins bind to endothelial ligands like ICAM-1 and VCAM-1, leading to the firm arrest of T cells on the endothelium, followed by migration into the tissue [88] (Fig. 2, 3).

The mechanisms behind T-cell trafficking between the thymus and secondary lymphoid organs, as well as the movement of DCs from the heart to the lymph nodes, remain largely unexplored. The heart is irrigated by lymph vessels, suggesting that this trafficking is essential for maintaining heart homeostasis [89].

Certain etiologies for heart failure, like myocarditis and in patients with heart transplant rejection, involve circulating immune cells, especially T cells, that will circulate to the myocardium and result in a reduction of the left ventricular function. In consequence these conditions typically respond well to immunomodulating therapies. However, the majority of HF cases derive from other common pathophysiological stresses, such as pressure overload (from hypertension or valve disease, especially aortic stenosis) or ischemic disease, rather than primary autoimmune disorders [90].

In chronic ischemic cardiomyopathy, there's a systemic expansion of CD4 + and CD8 + T cells, as well as CD4 + T helper 1, T helper 2, T helper 17, and regulatory T (T reg) subsets in the failing heart, circulation, and lymphoid organs. Mice lacking Rag2, and therefore without functional B and T cells, were protected from the shift from hypertrophy to heart failure following transverse aortic constriction [91]. Autoreactive T helper cells, which target an antigen in cardiomyocytes, can drive the transition from hypertrophy to heart failure under pressure overload conditions. Following a myocardial infarction, CD4 + T cells have been noted to promote collagen matrix formation, aiding wound healing and enhancing survival by reducing the risk of myocardial rupture [91].

Patients with HF exhibit higher frequencies of proinflammatory CD4 + T helper 1 (Th1) and Th17 cells, and lower frequencies of regulatory T cells, with these features correlating with disease severity. Reducing T cell infiltration could be a novel treatment target in HF [92]. CD8 + lymphocyte depletion is independently linked to death, decreased 6-min walk distance, and increased New York Heart Association (NYHA) classification [93]. This depletion is found in some patients suffering from arterial pulmonary hypertension and worsens as the disease progresses. Additionally, T reg cell deficiency is associated with the progression of pulmonary hypertension [94].

### B Cells

Recent experimental and clinical observations suggest a link between the activation of humoral immune responses following myocardial heart failure. Studies using RAG2/SCID mouse models show that pathways leading to B cell activation play a key role in heart failure and disease progression [95]. Mice lacking programmed cell death protein-1, crucial for B cell differentiation, develop severe spontaneous dilated cardiomyopathy. High levels of circulating IgG bind specifically to cardiac myocytes. Interaction of B cells with T helper 1 stimulates cytokine production, affecting contractility and adverse remodeling [96] (Fig. 3).

Activated B cells can induce apoptosis of myocytes through complement-mediated cytotoxicity. In cases of multiple myocardial infarctions, the immune system’s encounter with myocardial proteins like troponin can lead to a persistent inflammatory state, enhancing myocardial cell death and injury [97]. In mouse models of ischemic cardiomyopathy, cytokine, IgM, and IgG expression increased threefold in the post-ischemic state compared to controls [98].

The presence of immunoglobulin IgG3 in both ischemic and nonischemic heart failure (HF) patients highlights a role for B cells in HF progression. In patients with advanced disease, there are increased levels of activated complement components in the circulation and, crucially, in the failing myocardium [99].

Limited clinical observations suggest that antibody removal strategies could influence HF progression. Overall, evidence indicates that following myocardial injury, B cell activation triggers downstream effects, leading to antimyocardial antibody formation, complement deposition, and additional myocardial injury [100].

## Angiotensin‑converting enzyme 2 and renin–angiotensin–aldosterone system biomarkers

Low cardiac output leading to reduced blood flow to the kidneys stimulates the overactivation of the renin–angiotensin–aldosterone system (RAAS). This process begins with the kidney's juxtaglomerular cells secreting more renin. Plasma renin converts angiotensinogen released by the liver into angiotensin I, which is further converted to angiotensin II. Angiotensin II, acting through AT1 receptors, results in the increased secretion of aldosterone from the adrenal cortex's zona glomerulosa. Aldosterone then activates mineralocorticoid receptors in kidney cells, leading to sodium and water retention and the excretion of potassium and magnesium. This increase in fluid volume within and outside blood vessels is central to the progression of heart failure [101].

Angiotensin II (Ang II) is known for its pro-inflammatory and pro-fibrotic characteristics and has been definitively linked to the development of HF, both with reduced and preserved ejection fraction. Angiotensin-converting enzyme 2 (ACE2) metabolizes Ang II into angiotensin 1–7 (Ang 1–7), which has potent anti-inflammatory, antioxidant, anti-fibrotic, and vasodilatory properties. Despite optimal Renin-Angiotensin System (RAS) blockade with ACE inhibitors, elevated levels of Ang II are associated with increased mortality and HF [102] (Fig. 1).

An increase of ACE2 activity has been linked to a rise in the severity HF and a decrease in left ventricular ejection fraction [103]. Wang et al. conducted a study where they measured plasma angiotensin peptides in a diverse group of HF patients. In a multivariate analysis adjusted for various factors, it was found that Ang 1–7/Ang II ratios above the median independently predicted lower mortality rates and shorter hospital stays. However, individual levels of Ang 1–7 or Ang II did not have any prognostic significance. The authors of the study interpreted this as an indication of the dynamic nature of the Renin-Angiotensin System (RAS) and its harmful effects due to an increase in Ang II levels without a corresponding increase in Ang 1–7 levels [104].

Aldosterone, a potent mineralocorticoid plays a multifaced role in promoting heart failure with reduced ejection fraction by influencing the effect of Ang II on plasminogen activator inhibitor-1. This, in turn, leads to increased oxidative stress and fibrosis at the organ level [35]. At the vascular level, excess aldosterone causes endothelial dysfunction, promotes the infiltration of inflammatory cells, accelerates the development of atherosclerosis and leads to plaque instability, arterial stiffness, and calcification. In the heart, aldosterone promotes inflammation, fibrosis, and myocardial hypertrophy. Clinically, high aldosterone levels are linked to a greater risk of cardiovascular events and mortality, particularly when aldosterone secretion is mismatched with renin levels and sodium intake, as seen in primary aldosteronism [105].

In vitro studies have demonstrated that aldosterone stimulates the synthesis of collagen by fibroblasts. Through the MAPK (mitogen-activated protein kinases) cascade [106], aldosterone also promotes cardiomyocyte hypertrophy [107], cardiac myofibroblast proliferation, and increases the release of matrix metalloproteinase by cardiomyocytes [108].

Mineralocorticoid receptor antagonists (MRAs) have been studied in patients with various forms of heart failure. For patients with HFrEF, the RALES (Randomized Aldactone Evaluation Study) [109] and EPHESUS (Eplerenone Post-Acute MI Heart Failure Efficacy and Survival Study) [110] trials showed that adding long-term treatment with 25 mg of spironolactone or up to 50 mg of eplerenone to standard therapy significantly reduces overall and cardiovascular mortality. In patients with HFpEF, spironolactone improved diastolic function but had mixed results on exercise capacity. The TOPCAT (Treatment of Preserved Cardiac Function Heart Failure With an Aldosterone Antagonist) trial found that spironolactone treatment in patients with HF and an ejection fraction of ≥ 45% did not reduce the primary composite outcome of cardiovascular mortality, aborted cardiac arrest, or hospitalization for HF, but did significantly reduce HF hospitalization [111].

## Primers of inflammation

### Myeloperoxidase

Myeloperoxidase (MPO) is a heme-containing enzyme primarily found in neutrophils. MPO-mediated oxidative stress can activate various inflammatory pathways, leading to the recruitment and activation of other immune cells, such as monocytes [112] (Fig. 1, Fig. 3).

MPO utilizes H2O2 produced by leukocyte or vascular NADPH oxidases to create various oxidizing molecules, including hypochlorous acid (HClO), chloramines, tyrosyl radicals, and nitrogen dioxides [113]. These MPO-derived reactive oxygen species (ROS) and reactive nitrogen species (RNS) not only have bactericidal effects but also cause tissue damage in the cardiovascular and renal systems and the brain [113].

MPO significantly influences vascular tone and endothelial NO availability, playing a role in atherogenesis, cardiovascular disease and contributes to myocardial dysfunction [113]. It plays a role in the development of both acute and chronic vascular inflammation, which is suggested to contribute to the pathogenesis of HFpEF [114].

Experimental studies with MPO knockout mice or oral MPO inhibitors showed reduced left ventricle dilation and improved left ventricular function in myocardial infarction models, indicating MPO's role in developing chronic heart failure [115]. In humans, CHF patients show higher systemic MPO levels, which are associated with poorer outcomes [116]. In cases of acutely decompensated CHF, higher MPO concentrations were linked to an increased risk of 1-year mortality [117].

The SATELLITE trial (Safety and Tolerability Study of AZD4831 in Patients with Heart Failure) investigated the MPO inhibitor AZD4831 (Mitiperstat) in patients with heart failure [118]. Although the trial was stopped early after achieving its initial goal of target engagement and demonstrating a satisfactory safety profile, it showed a 69% decrease in MPO activity within 30 days, along with improvements in exercise capacity and wellness scores [118]. This suggests that MPO inhibition could be a potential treatment for HFpEF. However, since MPO inhibition is a novel approach for treating HFpEF, further studies and trials are necessary to enhance our understanding and investigate the efficacy of this treatment [23].

### NLRP3 inflammasome

Inflammasomes are multiprotein complexes that act as molecular platforms to activate caspase-1 and other caspases, regulating the maturation of powerful proinflammatory cytokines such as IL-1β and IL-18 [119]. This process ultimately facilitates an inflammasome-mediated immune response. Among inflammasomes, the NLRP3 (nucleotide-binding domain, leucine-rich–containing family, pyrin domain–containing-3) inflammasome has received the most attention and is the best-characterized member of the NLR family [120] (Fig. 3). NLRP3 serves as the primary inflammasome sensor in cardiovascular diseases. When exogenous pathogen-associated molecular patterns or endogenous damage-associated molecular patterns are recognized, the activation of the NLRP3 inflammasome leads to inflammatory responses in various diseases, particularly chronic inflammatory disorders [120]. The activation of the NLRP3 inflammasome is involved in several pathological processes and conditions, including obesity, diabetes mellitus, gout, rheumatoid arthritis, tumors, subarachnoid hemorrhage, neurodegenerative disorders, Crohn's disease, chronic obstructive pulmonary disease, asthma, cryopyrin-associated periodic syndrome, geographic atrophy, and sepsis [121].

### Gut microbiota

The intestine serves as the largest interface between the internal body and the external environment. The intestinal barrier is a dynamic system influenced by the makeup of the intestinal microbiome and the activity of intercellular connections, which are regulated by hormones, dietary components, inflammatory mediators, and the enteric nervous system [122]. The human intestine hosts numerous microorganisms, collectively known as the"gut microbiota,"which consists of around ${10}^{13}$ bacterial cells. This microbiota includes over 250 species of viruses, fungi, bacteria, and archaea. It is a dynamic system that evolves throughout a person's life [123].

Lipopolysaccharides (LPS) also known as endotoxins are a part of the outer membrane of the Gram-negative bacteria and have pro-inflammatory properties. Under normal conditions, the gut barrier (comprising the intestinal epithelial and mucosal layers) minimizes the movement of LPS from the intestines into the bloodstream. When the intestines permeability is dysregulated macrophages can infiltrate the area, produce, and activate inflammatory cytokines, resulting in local inflammation [124]. LPS can harm the gut by causing inflammation and disrupting tight junctions between cells, leading to oxidative stress and other cellular damage [125]. This dysregulated permeability of the gut means macrophages can infiltrate the region, produce and activate inflammatory cytokines, leading to local inflammation [126]. LPS indirectly triggers inflammation by binding to Toll-Like Receptor 4 (TLR4) on various immune and non-immune cells [127]. These pathways activate different pro-inflammatory cytokines through the NF-kB transcription factor pathway and other pathways involving mitogen-activated protein kinases [128].

The relevance of LPS translocation has been studied in HF, where it is associated with worsened cachexia due to the secretion of pro-inflammatory cytokines. These patients were found to have increased intestinal permeability, leading to the translocation of bacteria and endotoxins that fuel the pro-inflammatory state [123].

### Mitochondrial dysfunction

The heart, being the most energetically active organ, houses more mitochondria than any other organ. Mitochondrial dysfunction can significantly affect the heart's energy status and lead to various pathological conditions. Such abnormalities can cause cardiomyocyte injury and death, generate reactive oxygen species (ROS), and impair cardiac cell function (Fig. 3). Additionally, mitochondria play crucial roles in cellular calcium homeostasis, vascular smooth muscle function, myofilament integrity and cell differentiation [129]. These elements are involved in the pathophysiology of heart failure, especially those in relation to calcium (Ca2 +) homeostasis and oxidative stress by diminishing the heart's contractile capacity, triggering excessive inflammatory responses, and interfere with the genetically programmed regulation of cell death [130].

ROS production maintains intracellular oxidative balance, but increased oxidative stress can lead to cellular inflammation and programmed cell death. This oxidative stress, driven by the overproduction of ROS (including free radicals and non-radical intermediates) contributes to cardiovascular disease [131]. In HFrEF, ROS are mainly produced by damaged cardiomyocytes, leading to remodeling through cell death and fibrosis [132]. Conversely, in HFpEF, ROS are predominantly generated by endothelial cells [3]. In both cases, mitochondria are the primary sources of reactive oxygen species [133].

### Endothelial inflammation

The endothelium, a single layer of cells lining the interior surface of blood vessels, serves as a critical functional and structural barrier between the circulating blood and the vessel wall. It prevents the adhesion and aggregation of platelets and leukocytes, regulates the permeability of plasma components, and modulates blood flow. Additionally, the endothelium exerts antiproliferative and anti-inflammatory effects, protects against oxidative stress, and maintains a balance between cellular proliferation and cell death [134].

Endothelial dysfunction reflects a shift in endothelial behavior, involving several maladaptive changes in its phenotype. These alterations disrupt the regulation of hemostasis and thrombosis, disturb vascular tone and redox balance, and lead to inflammatory dysregulation [135]. Endothelial dysfunction is recognized as a contributing factor in heart failure, and exploring the underlying mechanisms that connect these conditions remains a crucial area of research.

### Chronic vs Acute heart failure

Patients with chronic heart failure frequently exhibit marked systemic vasoconstriction and reduced peripheral tissue perfusion. Endothelial dysfunction is a key contributor to this state, as it intensifies preexisting vasoconstriction and augments myocardial injury by increasing afterload through both systemic and pulmonary vascular constriction. It also underlies the regional dysregulation of vasomotor tone in the renal and coronary circulations. The diminished coronary endothelium-dependent vasodilation impairs myocardial perfusion, reduces coronary flow, and worsens ventricular function. Although the resulting decrease in cardiac output normally raises endothelial shear stress to trigger eNOS expression, in heart failure eNOS becomes down-regulated, leading to decreased nitric oxide production and reduced flow-mediated vasodilation, thus favoring further vasoconstriction. Moreover, increased production of vasoconstrictors (primarily endothelin-1) exacerbates vascular resistance, stimulates smooth muscle cell proliferation and matrix deposition, and ultimately drives vascular remodeling, endothelial dysfunction, and the progression of heart failure [136, 137] (Fig. 2).

There is a hypothesis suggesting that acute heart failure arise as a consequence of acute endothelitis. According to this theory, an inflammatory trigger, such as an infection or medication noncompliance, can initiate systemic endothelitis, a condition marked by endothelial oxidative stress and activation [138]. This cascade sets off a series of vascular, renal and neurohormonal responses that promote fluid retention. Furthermore, congestion and the subsequent activation of a stretched endothelium further exacerbate systemic endothelitis, leading to the constriction of capacitance veins and the centralization of blood. This centralization impairs cardiac function and establishes a vicious cycle, where ongoing fluid retention ultimately results in clinical decompensation [139]. As for hemodynamics, sodium excretion is regulated by both arterial and venous pressures. When arterial filling is compromised, vascular and neurohumoral responses (such as sympathetic nervous system activation, catecholamine release and RAAS stimulation) are triggered, leading to fluid retention [140]. Additionally, acute heart failure is marked by an increase in oxidative stress, where excessive reactive oxygen species bind to nitric oxide, thereby reducing its bioavailability [140].

### HFrEF vs HFpEF

HFrEF worsen endothelial dysfunction through neurohumoral activation and increased shear stress. This condition elevates the synthesis of reactive oxygen species while reducing nitric oxide production [141]. The resulting imbalance between NO and oxidative stress impairs endothelium-dependent vasodilation, a change that is especially pronounced in coronary vessels, leading to reduced myocardial perfusion and compromised ventricular function. Over time, this process contribute to the progression of chronic heart failure [141].

Several studies have demonstrated that HFpEF is associated with endothelial damage, likely stemming from a systemic inflammatory state [142, 143]. In this context, cytokine production (particularly TNF-alpha) induces the transformation of endothelial cells into fibroblast-like cells while downregulating eNOS expression, a process that correlates with the severity of endothelial dysfunction [144]. This cascade reduces cyclic-guanosine monophosphate (c-GMP) levels, leading to microvascular ischemia, increased diastolic cytosolic calcium, and impaired myocardial relaxation [145]. Additionally, the dysfunction of matrix metalloproteinases results in the accumulation of extracellular matrix proteins, fostering cardiac hypertrophy, fibrosis, and myocardial stiffness, and ultimately progressing to diastolic dysfunction [146].

## Novel anti-inflammatory strategies in heart failure

### Approved treatments

#### Colchicine (Figure 3)

Colchicine, an anti-inflammatory agent often used for conditions like gout, pericarditis, and Behcet's syndrome, works by blocking the activation of the NLRP3 inflammasome. This inhibition reduces the production of IL-1 beta and IL-18, prevents tubulin polymerization and microtubule development, and impairs neutrophil migration. Consequently, it inhibits IL-1 production by activated neutrophils and downregulates TNF alpha receptors in macrophages and endothelial cells [147].

The use of colchicine for chronic cardiovascular disease was investigated in the COLCOT trial, which involved 4,745 post-myocardial infarction individuals randomized to receive either colchicine or a placebo. The study found a 23% relative risk reduction in the primary cardiovascular endpoint. However, only 2% of the study population had a history of heart failure [148].

A study that shows the impact of colchicine in patients with HF was the COLICA trial that included 279 patients that were enrolled for a period of 4 years in which they aimed to demonstrate that colchicine was safe and effective in reducing inflammation markers in patients with HF regardless of their left ventricular ejection fraction. The main outcome, which was the average reduction in NT-proBNP levels over 8 weeks, did not differ between the colchicine and placebo groups. However, colchicine led to a significant decrease in inflammatory markers such as C-reactive protein and interleukin-6. There were no notable differences in the incidence of new worsening heart failure episodes, although the requirement for intravenous furosemide was less in the colchicine group [149].

#### Statins

Statins not only lower cholesterol but also have numerous pleiotropic effects, including anti-inflammatory properties that reduce CRP levels by 15%−30%. They improve endothelial function by inducing eNOS, inhibit adhesion molecules like VCAM-1 and ICAM-1, reduce the effect of NFKB, and disrupt T cell activation (Fig. 2) [150]. Observational studies and post-hoc subgroup analyses have suggested that statins might improve outcomes in HF patients [151, 152]. However, large clinical randomized trials involving statin use in HFrEF have shown no benefit [153]. Many HFpEF patients are likely to be prescribed statins, for example, for coronary artery disease. For instance, almost 70% of patients in the EMPEROR-Preserved trial were taking statins at baseline [154].

#### Glucagon-like peptide-1 (GLP-1) receptor agonist – Semaglutide

Inflammation seems to play a key role in the development of obesity [155] that is an independent factor for cardiovascular events. Semaglutide, a GLP-1 receptor agonist, has been efficient in lowering the risk of adverse cardiovascular events in diabetic patients [156].

SELECT Trial was a randomized, placebo-controlled trial involving patients with preexisting cardiovascular disease and overweight or obesity, but without diabetes, has proved that a weekly subcutaneous administration of semaglutide at a dose of 2.4 mg was more effective than placebo in decreasing the incidence of a composite of death from cardiovascular causes, nonfatal myocardial infarction, or nonfatal stroke over an average follow-up period of 39.8 months [157]. Starting from this a prespecified analysis has demonstrated that a treatment with semaglutide 2.4 mg once weekly significantly improved outcomes in heart failure patients across various baseline characteristics (age, sex, adiposity measures, glycemic control, lipids and blood pressure). The observed reduction in all-cause mortality across all heart failure groups suggests the possibility of other, yet unidentified, benefits [158].

The STEP-HFpEF Program was a second analysis from 2 placebo-controlled trials (STEP-HFpEF and STEP-HFpEF DM) that randomized a total of 1,145 patients, 71% of whom had evidence of inflammation (CRP ≥ 2 mg/L). Compared to placebo, semaglutide resulted in reductions in HF-related symptoms and physical limitations, as well as in body weight, and improvements in 6-m walking test. Semaglutide also reduced CRP more effectively than placebo, regardless of initial CRP levels and changes in CRP from baseline to 52 weeks were similar irrespective of the degree of weight loss [159].

#### Finerenone

Finerenone targets mineralocorticoid receptors (MRs), which are widely distributed in the heart, blood vessels, and principal kidney cells. The primary advantage of finerenone is thought to be its ability to reduce inflammation and fibrosis in the heart and kidneys. Another advantage over other MRAs is its high selectivity for the mineralocorticoid receptor [160]. This effect occurs by blocking the downstream mechanisms triggered by aldosterone's activation of the MR. MRs in various cell types, such as cardiomyocytes and myeloid cells, which are implicated in the inflammation and fibrosis of the heart and vascular system, contributing to heart failure [161].

The FIDELIO-DKD and FIGARO-DKD trials demonstrated that finerenone, when added to a renin–angiotensin system blockade or standard therapy, effectively reduces cardiovascular outcomes compared to a placebo added to the same treatments [160, 162]. The incidences of all HF outcomes analyzed were significantly lower with finerenone than placebo in the FIGARO-DKD trial. This suggests that finerenone has positive effects on heart failure in patients with chronic kidney disease and type 2 diabetes mellitus [160]. This conclusion is further supported by the reduction in the key secondary composite outcome (death from cardiovascular causes, nonfatal myocardial infarction, nonfatal stroke, or hospitalization for heart failure) observed in the FIDELIO-DKD trial [162].

An ongoing program for finerenone in patients with heart failure, the MOONRAKER includes four studies: FINEARTS-HF, REDEFINE-HF, CONFIRMATION-HF and FINALITY-HF [163]. The FINEARTS-HF was completed in August 2024 and had a composite of cardiovascular death and total worsening HF events as primary outcome and demonstrated that finerenone, when added to standard therapy, was effective and well-tolerated in patients with heart failure and a left ventricular ejection fraction (LVEF) of 40% or higher (HFmrEF/HFpEF) [164, 165]. REDEFINE-HF is a phase III study that will investigate the efficacy and safety of finerenone compared to placebo in addition to standard of care in reducing total heart failure events and cardiovascular death in patients with decompensated heart failure with an ejection fraction of ≥ 40% (HFmrEF/HFpEF) [166]. CONFIRMATION-HF will investigate finerenone in addition to an SGLT2 inhibitor compared to standard of care in patients with heart failure regardless of left ventricular ejection fraction [167]. FINALITY-HF is another phase III randomized, double-blind, placebo-controlled, parallel-group, multicenter, event-driven study that will evaluate the efficacy and safety of finerenone in addition to standard of care compared to placebo in reducing cardiovascular death or heart failure events in patients with heart failure with an ejection fraction < 40% (HFrEF) who are intolerant or not eligible for steroidal MRAs like spironolactone or eplerenone [168].

### Experimental therapies

#### Methotrexate (Figure 3)

Methotrexate, commonly used for conditions like rheumatoid arthritis (RA), has been linked to a reduction in cardiovascular events among patients with rheumatological conditions. A recent retrospective study involving 9,889 patients with rheumatoid arthritis and matched controls found that this treatment was associated with a lower risk of incident HF with preserved ejection fraction. This suggests a potential role for methotrexate in managing inflammation in HFpEF, especially in patients with a high inflammatory profile [169].

In the Cardiovascular Inflammation Reduction Trial (CIRT), individuals with a history of MI or coronary artery disease and either type 2 diabetes or metabolic syndrome were randomized to receive low-dose methotrexate (up to 20 mg daily) or placebo. Around 13% of participants had a history of HF. After a median follow-up of 2.3 years, methotrexate did not reduce levels of IL6, IL1beta, and CRP, nor did it decrease the incidence of the primary endpoint of cardiovascular death, myocardial infarction, or stroke. In a small, randomized trial involving 71 HF with reduces ejection fraction patients, 12 weeks of methotrexate treatment reduced inflammatory markers, improved NYHA class, quality of life, and increased 6-min walk distance, without changing LV ejection fraction [170].

#### Interleukin-1 Receptor Antagonist – Anakinra (Figure 3)

In the DHART trial, a 2-week treatment with anakinra, an IL-1 receptor antagonist, reduced systemic inflammation and improved aerobic exercise tolerance and peak Vo_2_ in patients with HFpEF [171]. In the larger follow-up study DHART2, although Anakinra reduced CRP and NTproBNP levels and improved exercise tolerance, it did not improve the primary endpoint of peak Vo_2_. The discrepancy in results be partially due to the fact that most participants in DHART2 were obese, affecting their cardiorespiratory fitness independently of cardiac function [172].

The REDHART Trial (Recently Decompensated Heart Failure Anakinra Response Trial) published in 2017 examined the impact of anakinra (100 mg once daily for 2 weeks or 12 weeks) versus placebo on exercise capacity in 60 patients with recently decompensated heart failure. Peak oxygen consumption (Vo_2_) and ventilatory efficiency (the VE/Vco2 slope) were measured. At 2 weeks, anakinra treatment did not impact peak Vo_2_ or VE/Vco2 slope. However, those who continued anakinra for 12 weeks showed an improvement in peak Vo2 [173].

#### Interleukin-6 Receptor Antagonist – Tocilizumab (Table 2, Figure 3)

**Table 2:** Results of studies on cardiovascular events after administration of Tocilizumab

STUDY AUTHOR	YEAR	AGE	GENDER	CV RF	N	FOLLOW-UP (YEAR)	MACE	MI	STROKE	HF
**BROCH** [178]	2021	62 ± 10	80% males	Smoker, DM, HTN	195	0.5	19	195	4	NS
**MEYER** [179]	2021	65	33% males	Smoker, HTN, DM	80	0.5	17	37	10	14
**KIM** [180]	2018	58,8 ± 10,4	81,4% females	HTA, Dyslipidemia, DM	6 237	NS	32	NS	NS	NS
**BURMESTER** [181]	2017	50.2 ± 13.6	77% females	NS	1 108	2	NS	NS	NS	NS
**CHOY** [182]	2017	54.3 ± 12.8	NS	NS	423	1	0	NS	0	NS
**IANNONE** [183]	2017	54.5	NS	HTA, DM, smoking	202	2	7	NS	NS	NS
**KIM** [184]	2017	58.9 ± 10,2	88,3% females	HTA, DM	9 218	NS	43	21	23	NS
**GILES** [185]	2016	61	78% females	HTA, DM, smoking	1538	3.2	83	29	26	12
**GOTTENBERG** [186]	2016	NS	NS	NS	NS	2	NS	NS	NS	NS
**ZHANG** [187]	2016	63.7	87% females	HTA, smoking, DM	3 332	NS	17	17	NS	NS
**CURTIS** [188]										
Insurance claims databases	2015	NS	NS	NS	62	NS	206	115	91	NS
Safety database	2015	NS	NS	NS	5 734	NS	28	14	14	NS
**SAKAI** [189]	2015	59.2 ± 13	82.5% females	DM	302	1	NS	NS	NS	NS
**GABAY** [190]	2013	54.4 ± 13	79% females	NS	162	0.5	3	2	1	NS
**SMOLEN** [191]	2008	51.1 ± 12.3	83.5% females	NS	418	1	29	NS	NS	NS

*NS* not specified, *HTN* hypertension, *DM* diabetus mielletus, *CV FR* cardiovascular risk factors, *MACE* major adverse cardiac events, *MI* myocardial infarction, *HF* heart failure.

In a cohort study involving 1,584 patients with RA unresponsive to TNF-α inhibitors, second-line treatment with tocilizumab significantly reduced the risk of MI by 88% and MACE by 59% compared to rituximab [174]. A recent network meta-analysis, combining data from both RCTs and observational studies of RA patients, showed that tocilizumab has a clear cardiovascular safety profile compared to other RA treatments and reduce the risk of MI compared to abatacept [175]. Another relevant meta-analysis indicated that tocilizumab could decrease the risk of MACE in RA patients compared to TNF inhibitors, although this finding was marginally non-significant [176].

Real-world data suggests that tocilizumab benefit cardiac function in RA patients without pre-existing CVD. An earlier observational study involving 20 female RA patients, and 20 age-matched healthy controls showed that after 52 weeks of tocilizumab treatment, RA patients experienced a significant increase in LVEF, a significant decrease in LV mass index, and normalization of baseline eccentric LV hypertrophy [177].

In another observational study involving 70 RA patients without pre-existing CVD, a 24-week treatment with tocilizumab significantly decreased NT-proBNP levels. This decrease strongly correlated with the observed changes in disease activity [70].

#### Human monoclonal antibody against Interleukin-6 – Ziltivekimab (Figure 3)

A fully human monoclonal antibody against interleukin-6 ligand called ziltivekimab was introduced in 2021 in the RESCUE trial. This trial focused on patients with elevated high-sensitivity C-reactive protein (hsCRP) and chronic kidney disease (CKD) and demonstrated that ziltivekimab significantly reduced hsCRP levels compared to placebo, with reductions of 77%, 88%, and 92% across different doses. This reduction was greater than that achieved in the CANTOS trial with IL-1β inhibition [192]. Additionally, ziltivekimab effectively decreased other inflammatory markers without increasing atherogenic lipids or causing serious side effects [193]. Based on these findings, an ongoing trial, ZEUS trial (Ziltivekimab Cardiovascular Outcomes Study), aims to test whether IL-6 inhibition with ziltivekimab can reduce major cardiovascular events in patients with CKD and elevated hsCRP levels and assess its impact on renal disease progression. Moreover, initial assessments of left ventricular ejection fraction will be conducted in the ZEUS trial to allow the stratification of participants based on heart failure phenotype with either reduced or preserved ejection fraction [194].

## Future directions

Inflammation plays a crucial role in the etiology and progression HF, yet there are significant gaps in our understanding of this complex relationship.

One of the areas that require further exploration is the inflammatory activation cascade, which leads to cardiac injury that concludes in increased hospitalization rates in chronic HF. While we have some understanding of the role of specific cytokines in this process, more research is needed to identify independent risk factors for HF decompensation or cardiovascular mortality and morbidity [195].

Another area that needs further investigation is the role of inflammation in different phenotypes of HF. Past studies have suggested that inflammation be triggered in varying amounts depending on the phenotype of HF [196]. However, the mechanisms underlying these differences remain unclear. Future research should aim to elucidate these mechanisms, which could lead to the development of phenotype-specific therapeutic strategies.

There is a need for more clinical trials evaluating specific cytokines and other potential therapeutic targets for inflammation in HF. Future research could also explore the potential benefits of existing guideline-directed medical therapies for heart failure on inflammatory pathways [35] (Table 3).

**Table 3:** Ongoing trials for Anti-inflammatory Medications in Heart Failure

STUDY	INTERVENTION	STUDY ENDPOINTS	ESTIMATED NUMBER OF PATIENTS	STUDY COMPLETION (Estimated)	INCLUSION CRITERIA
**ENDEAVOR** **(NCT04986202)**[197]	Mitiperstat (AZD4831) 2.5 or 5 mg daily for 48 weeks	Change in 6-min walk distance and QoL at 16 week	711	27.03.2024—completed	Symptomatic HFpEF/HFmrEF with elevated BNP/NT-proBNP
**Colchicine in HFpEF (NCT05637398)**[198]	Colchicine, 0.5 mg BID for 12 weeks	Change in hsCRP, NT-proBNP, and Doppler echocardiographic parameters after 12 weeks	40	01.11.2024—completed	Stable symptomatic HFpEF with BMI > 30 kg/m2 or DM
**AID-HEART** **(NCT06062966)**[199]	Anakinra, 100 mg daily	Change in hsCRP, inotrope dose use, and 6-min walk test at 1 and 3 months	20	08.2025	End-stage HFrEF on chronic stable inotrope therapy with hsCRP > 2 mg/L
**Colchicine in ADHF** **(NCT06286423)**[198]	Colchicine, 0.6 mg BID for 14 days, then daily for another 76 days	Change in hsCRP at 3 and 14 days; all-cause death or HF hospitalization at 90 days	30	30.06.2026	Acutely decompensated HFrEF with hsCRP > 2 mg/L and elevated BNP/NT-proBNP
**ATHENA** **(NCT06200207)**[200]	Ziltivekimab, 15 mg once monthly for 12 months	Change in KCCQ, 6-min walk distance, NYHA class, hsCRP, NT-proBNP, and renal function at 12 months	680	05.10.2026	Symptomatic HFpEF with hsCRP > 2 mg/L and elevated NT-proBNP
**HERMES** **(NCT05636176)**[200]	Ziltivekimab, 15 mg once monthly up to 4 years	Time to first occurrence CV death, HF hospitalization or urgent HF visit, nonfatal MI, and nonfatal stroke up to 48 months	5600	02.07.2027	Symptomatic HFpEF with hsCRP > 2 mg/L and elevated NT-proBNP

*Qol* quality of life, *CV* cardiovascular, *KCCQ* Kansas City Cardiomyopathy Questionnaire, *MI* myocardial infarction, *BMI* body mass index, **ENDEAVOR –** Study to Evaluate the Efficacy and Safety of AZD4831 in Participants With Heart Failure With Left Ventricular Ejection Fraction > 40%, **AID-HEART** – The Effects of IL-1 Blockade on Inotrope Sensitivity in Patients With Heart Failure, **ADHF –** acute decompensated heart failure, **ATHENA –** A Research Study Looking Into How Ziltivekimab Works Compared to Placebo in Participants With Heart Failure and Inflammation, **HERMES –** A Research Study to Look at How Ziltivekimab Works Compared to Placebo in People With Heart Failure and Inflammation.

## Conclusion

This review provides a detailed analysis of the role inflammation has in the development and progression of heart failure and highlights the complexity of pro-inflammatory pathways and their contribution to the pathogenesis of HF.

The understanding of the inflammation implication in the etiology of HF could guide the development of novel therapies. The review also discusses established and novel biomarkers of inflammation and how they can be used to understand the role of inflammation in HF.

## Data Availability

No datasets were generated or analysed during the current study.
